# Supplementary material for: Automated Detection of Gibbon Calls From Passive Acoustic Monitoring Data Using Convolutional Neural Networks in the “Torch for R” Ecosystem
Source: Ecol Evol. 2025 Jul 14;15(7):e71678. doi: 10.1002/ece3.71678 (PMC12259389; doi:10.1002/ece3.71678)
Supplement: Supplementary file 1 — Appendix S1 [file ECE3-15-e71678-s001.docx]

**Appendix**

**Appendix Table 1. Results of the “Benchmarking Part 2” experiments comparing the performance of different CNN architectures trained for 1, 2, 3, 4, 5 and 20 epochs with early stopping.** For crested gibbons there were multiple combinations with high performance.

| Species | N epochs | CNN Architecture | Threshold | Precision | Recall | F1 | AUC |
| --- | --- | --- | --- | --- | --- | --- | --- |
| Crested Gibbon   binary | 1, 3, 4, 5 | resnet152 | 0.1, 0.2, 0.3, 0.4, 0.5, 0.6, 0.7, 0.8, 0.9 | 1.00 | 1.00 | 1.00 | 1.00 |
|  | 2, 20 | vgg19 | 0.4, 0.5, 0.6, 0.7, 0.8 | 1.00 | 1.00 | 1.00 | 1.00 |
|  | 3, 4, 5 | resnet18 | 0.1, 0.2, 0.3, 0.4, 0.5, 0.6, 0.7 | 1.00 | 1.00 | 1.00 | 1.00 |
|  | 3 | resnet50 | 0.4 | 1.00 | 1.00 | 1.00 | 1.00 |
|  | 5 | vgg16 | 0.9 | 1.00 | 1.00 | 1.00 | 1.00 |
| Grey Gibbon   binary | 4 | alexnet | 0.9 | 0.97 | 0.93 | 0.95 | 0.99 |
| Crested Gibbon   multi | 1, 2, 3, 4, 5, 20 | resnet18 | 0.1, 0.2, 0.3, 0.4, 0.5, 0.6, 0.7, 0.8, 0.9 | 1.00 | 1.00 | 1.00 | 1.00 |
|  | 1, 3, 4, 5 | resnet50 | 0.1, 0.2, 0.3, 0.4, 0.5, 0.6, 0.7, 0.8, 0.9 | 1.00 | 1.00 | 1.00 | 1.00 |
|  | 4, 20 | resnet152 | 0.1, 0.2, 0.3, 0.4, 0.5, 0.6, 0.7, 0.8, 0.9 | 1.00 | 1.00 | 1.00 | 1.00 |
|  | 4, 5, 20 | vgg16 | 0.1, 0.2, 0.3, 0.4, 0.5, 0.6, 0.7, 0.8, 0.9 | 1.00 | 1.00 | 1.00 | 1.00 |
|  | 4, 5, 20 | vgg19 | 0.2, 0.3, 0.4, 0.5, 0.6, 0.7, 0.8, 0.9 | 1.00 | 1.00 | 1.00 | 1.00 |
|  | 4 | alexnet | 0.4 | 1.00 | 1.00 | 1.00 | 1.00 |
| Grey Gibbon   multi | 2 | resnet50 | 0.6 | 0.95 | 0.96 | 0.95 | 0.99 |

**
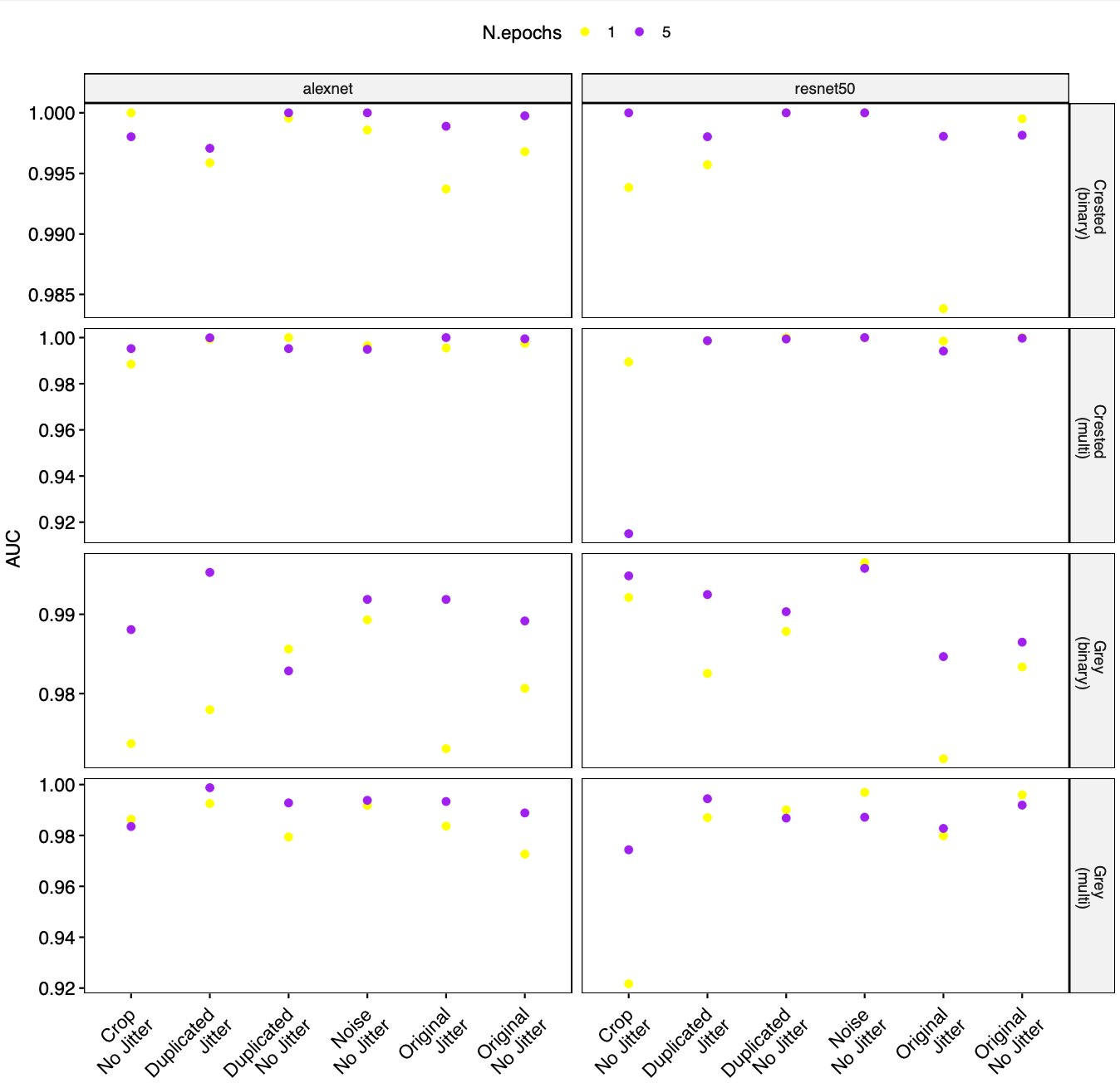
**

**Appendix Figure 1. AUC−ROC scores across training data augmentation types and architectures for “Benchmarking Part 3”.** AUC−ROC was calculated on the original test data split. The color of the points indicates if the model was trained for one epoch (yellow) or five epochs (purple).

**
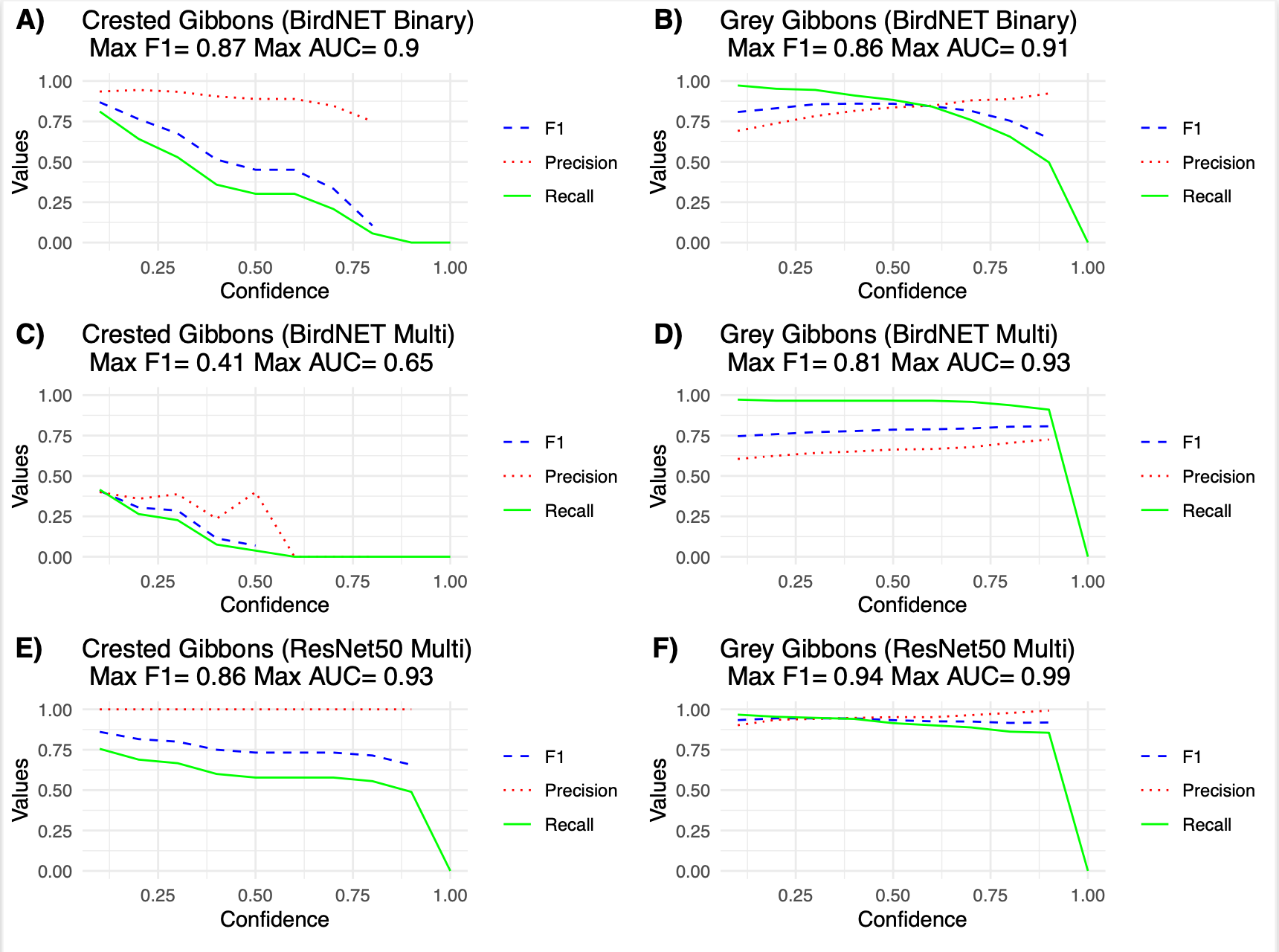
**

**Appendix Figure 2. Precision, recall, and F1 score as a function of confidence score for BirdNET (binary and multiclass) and ResNet50 (multiclass only) models for classification of gibbon calls.** BirdNET models were trained on the original training data, and multiclass ResNet50 models were trained on the “duplicated” dataset with color jitter.
